# Serum Soluble Mediator Profiles and Networks During Acute Infection With Distinct DENV Serotypes

**DOI:** 10.3389/fimmu.2022.892990

**Published:** 2022-05-31

**Authors:** Mikelly Santos Coutinho-da-Silva, Pedro Henrique Ferreira Sucupira, Kelly Alves Bicalho, Ana Carolina Campi-Azevedo, Joaquim Pedro Brito-de-Sousa, Vanessa Peruhype-Magalhães, Maria Rios, Andréa Teixeira-Carvalho, Jordana Grazziela Alves Coelho-dos-Reis, Lis Ribeiro do Valle Antonelli, Vitor Bortolo de Rezende, Fernanda Ludolf Ribeiro de Melo, Cristiana Couto Garcia, Jesuanne Carla Silva-Andrade, Ismael Artur da Costa-Rocha, Michele de Souza Bastos, Lucia Alves da Rocha, Valderjane Aprigio Silva, Ewerton da Silva Ferreira, Eveny Perlize Melo Marinho, Allyson Guimarães Costa, Matheus de Souza Gomes, Laurence Rodrigues Amaral, Erilene Cristina da Silva Furtado, Eliana Vieira Pinto da Silva, Bruna Alves Ramos, Éder Barros dos Santos, Maria Nazaré Oliveira Freitas, Pedro Fernando da Costa Vasconcelos, Olindo Assis Martins-Filho, Márcio Sobreira Silva Araújo, Milene Silveira Ferreira, Livia Carício Martins

**Affiliations:** ^1^Departamento de Arboviroses e Febres Hemorrágicas, Instituto Evandro Chagas, Ananindeua, Brazil; ^2^Instituto René Rachou, Fundação Oswaldo Cruz (FIOCRUZ-Minas), Belo Horizonte, Brazil; ^3^Office of Blood Research and Review (OBRR), Center for Biologics Evaluation and Research (CBER), U.S. Food and Drug Administration (FDA), Silver Spring, MD, United States; ^4^Departamento de Microbiologia, Instituto de Ciências Biológicas, Universidade Federal de Minas Gerais (UFMG), Belo Horizonte, Brazil; ^5^Faculdade de Medicina, Universidade Federal de Minas Gerais, Belo Horizonte, Brazil; ^6^Laboratório de Vírus Respiratórios e Sarampo, Instituto Oswaldo Cruz, Fiocruz, Rio de Janeiro, Brazil; ^7^Instituto de Pesquisa Clínica Carlos Borborema, Fundação de Medicina Tropical Dr. Heitor Vieira Dourado, Manaus, Brazil; ^8^Escola de Enfermagem de Manaus, Universidade Federal do Amazonas (UFAM), Manaus, Brazil; ^9^Diretoria de Ensino e Pesquisa, Fundação Hospitalar de Hematologia e Hemoterapia do Amazonas (HEMOAM), Manaus, Brazil; ^10^Laboratório de Bioinformática e Análises Moleculares, Rede Multidisciplinar de Pesquisa, Ciência e Tecnologia, Universidade Federal de Uberlândia (UFU), Patos de Minas, Brazil

**Keywords:** dengue infection, dengue virus, immune response, DENV serotypes, chemokines, cytokines

## Abstract

A panoramic analysis of chemokines, pro-inflammatory/regulatory cytokines, and growth factors was performed in serum samples from patients with acute DENV infection (n=317) by a high-throughput microbeads array. Most soluble mediators analyzed were increased in DENV patients regardless of the DENV serotype. The substantial increase (≥10-fold) of CXCL10, IL-6, and IFN-γ, and decreased levels of PDGF (<0.4-fold) was universally identified in all DENV serotypes. Of note, increased levels of CXCL8, CCL4, and IL-12 (≥3-9-fold) were selectively observed in DENV2 as compared to DENV1 and DENV4. Heatmap and biomarker signatures further illustrated the massive release of soluble mediators observed in DENV patients, confirming the marked increase of several soluble mediators in DENV2. Integrative correlation matrices and networks showed that DENV infection exhibited higher connectivity among soluble mediators. Of note, DENV2 displayed a more complex network, with higher connectivity involving a higher number of soluble mediators. The timeline kinetics (Day 0-1, D2, D3, D4-6) analysis additionally demonstrated differences among DENV serotypes. While DENV1 triggers a progressive increase of soluble mediators towards D3 and with a decline at D4-6, DENV2 and DENV4 develop with a progressive increase towards D4-6 with an early plateau observed in DENV4. Overall, our results provided a comprehensive overview of the immune response elicited by DENV infection, revealing that infection with distinct DENV serotypes causes distinct profiles, rhythms, and dynamic network connectivity of soluble mediators. Altogether, these findings may provide novel insights to understand the pathogenesis of acute infection with distinct DENV serotypes.

## Introduction

Dengue virus infection (DENV) is a mosquito-borne disease caused by one of the four dengue serotypes (DENV-1, DENV-2, DENV-3, and DENV-4) that belongs to the *Flaviviridae* family. DENV serotypes circulate concomitantly in different regions worldwide, covering approximately 100 countries in tropical and subtropical areas of the globe with expanding geographical range and incidence (1, 2). The precise incidence of DENV is difficult to determine but it is estimated that the annual number of cases ranges from 284 to 528 million worldwide (3) depending on the year and intensity of outbreaks.

DENV infection induces a spectrum of clinical manifestations, ranging from asymptomatic to life-threatening hemorrhagic fever/shock syndrome. The pathogenesis of DENV involves a complex interplay of viral and host factors, including viral serotype, host age, genetic background, and immunological status (4–7).

Previous studies have suggested that distinct serotypes may cause more severe disease. It has been shown that DENV-2 infection is associated with higher rates of severe clinical forms of DENV infections (8). It has been considered that different serotypes may vary in their ability to infect target cells, triggering distinct immune response profiles that, in turn, impact their capacity to cause severe clinical forms (9, 10). However, the precise mechanisms explaining the association between higher disease severity and distinct viral serotypes are still unclear.

Among the host factors, the immune responses are critical for the outcome of DENV infection. High levels of serum soluble mediators have been detected during DENV infection (11–16). The upregulated levels of chemokines and cytokines may contribute to both protective and immunopathological mechanisms during DENV infection (17). Moreover, higher plasma levels of pro-inflammatory mediators have been found in patients with severe dengue (18). It is believed that inflammatory cytokine storm and other soluble mediators can act on the endothelium, and alter normal fluid barrier functions, leading to increased plasma leakage (19, 20). However, much remains to be elucidated about the immunopathogenesis of acute DENV infections in humans, particularly focusing on the impact of distinct DENV serotypes during acute infection.

In the present study, a panoramic analysis of several chemokines, pro-inflammatory/regulatory cytokines, and growth factors was performed in serum samples from patients with acute DENV infection with distinct serotypes (DENV-1, DENV-2, and DENV-4) using a high-throughput microbeads array. Overall, our findings demonstrate that the profile of circulating soluble mediators differs substantially during acute DENV infection according to distinct serotypes. Selective profiles elicited in each response suggest the participation of distinct serotype-associated immune pathways that may ultimately represent a potential target for future therapeutic intervention.

## Material and Methods

### Subjects and Samples

This is a cross-sectional study submitted and approved by the Ethical Committee at Instituto René Rachou/Fundação Oswaldo Cruz (FIOCRUZ-Minas) – Belo Horizonte, Minas Gerais, Brazil (CAAE 41591015.2.0000.5091), and Fundação de Medicina Tropical Dr. Heitor Vieira Dourado, Manaus, Amazonas, Brazil (CAAE 80866017.8.0000.0005). A total of 317 patients with acute DENV infection, enrolled from January 2010 to December 2019, were selected for this investigation as a non-probabilistic convenience sample, comprising 153 males and 164 females, aging from 6 months to 74 years old (median = 27 years). Serum samples from DENV patients were obtained from biorepositories maintained at Instituto Evandro Chagas, Ananindeua, Pará, Brazil, and Fundação de Medicina Tropical Dr. Heitor Vieira Dourado, Manaus, Amazonas, Brazil. Demographic, clinical, and laboratory records were obtained from the compulsory notification form for DENV infection available at the Information System for Notifiable Diseases (SINAN) of the Ministry of Health of Brazil. Laboratory diagnosis of DENV infection was carried out by serological test (MAC-ELISA), molecular tests (RT-qPCR), or viral isolation in cell culture. Differential diagnosis with other acute febrile viral infections (ZIKV and CHIKV) was also carried out, and cases of co-infection were excluded from the present investigation. DENV serotype diagnosis was performed in 269 DENV patients by qPCR or indirect immunofluorescence test, allowing further classification of patients into three subgroups, referred to as DENV1 (n=116), DENV2 (n=52), and DENV4 (n=101). No DENV3 infection was detected amongst the patients enrolled in the present investigation. Days after symptom onset was available for 313 DENV patients and was used to cluster them into subgroups for a cross-sectional analysis at four consecutive time points (Days = D), including D0-1, D2, D3, and D4-6.

As a reference control group, a total of 319 age-matching healthy subjects were included, comprising 148 males and 171 females, aging from 1 to 75 years old (median = 20 years). The control group was composed of a non-probabilistic convenience sampling from biorepositories maintained at Grupo Integrado de Pesquisas em Biomarcadores, Instituto René Rachou, Fundação Oswaldo Cruz (FIOCRUZ-Minas), Belo Horizonte, MG, Brazil and at Fundação Hospitalar de Hematologia e Hemoterapia do Amazonas (HEMOAM), Manaus, Amazonas, Brazil.

All participants enrolled in the present investigation signed the informed consent form under the Declaration of Helsinki and Resolution 466/2012 of the Brazilian National Health Council for research involving human subjects.

### DENV Diagnosis

DENV diagnosis was performed by serological test, molecular tests, or viral isolation in cell culture, as described in detail below.

Serological diagnosis was performed by *in-house* IgM capture enzyme-linked immunosorbent assay for DENV (MAC-ELISA) as previously described (21). Briefly, flat-bottom 96-well plates were sensitized with goat anti-human IgM and blocked with 4% bovine serum albumin (BSA) solution. Plates were washed and incubated with 50 µL/well of serum samples for 2 hours. Following washing steps, 50 µL of soluble DENV antigen were added to each well, and plates were incubated at 4°C overnight. Plates were washed and 25 µL/well of anti-flavivirus monoclonal antibody (6B6C, Jackson Immunoresearch Laboratories, PA, USA) conjugated with horseradish peroxidase were added. Plates were washed and incubated with 100 µL/well of ABTS substrate (2.2’-azino-di-(3-ethyl-benzthiazoline sulfonate) and hydrogen peroxide. After incubation for 30 min at room temperature (RT), plates were read in an ELISA reader at 405 nm and positivity was assessed according to the results observed for negative control samples.

Molecular diagnosis was performed by reverse transcription-quantitative polymerase chain reaction (RT-qPCR) as described (22). Briefly, purified RNA was extracted from 200 μL of serum samples using the ReliaPrep™ Viral TNA Miniprep System (Promega, WI, USA) and then reverse transcribed to cDNA using SuperScript™ II Reverse Transcriptase (Invitrogen, USA). Experiment batches were carried out according to the manufacturer’s instructions. cDNA was used as a template for the qPCR employing the GoTaq® qPCR Master Mix Kit (Promega, WI, USA) for the first screening of Flavivirus using the following primers: mFU-1 (5’-TACAACATGATGGGAAAGCGAGAGAAAAA-3’), and CFD2 (5’-GTGTCCCAGCCGGCGGTGTCATCAGC-3’) (22). DENV molecular diagnosis was performed according to previous studies (23) using D1 (5’-TCAATATGCTGAAACGCGCGAGAAACCG-3’), and D2 (5’-TTGCACCAACAGTCAATGTCTTCAGGTTC-3’) primers.

DENV viral isolation was carried out in continuous C6/36 cell line cultures *in vitro* as described previously (24).

### DENV Serotype Diagnosis

Differential diagnosis of DENV serotypes (DENV1-4) was performed as previously described (23). Samples that tested positive for DENV by RT-qPCR were submitted to a second round of amplification using 10 μL of the first reaction with a mix of DENV serotype-specific reverse primers (TS1 = 5’-CGTCTCAGTGATCCGGGGG-3’; TS2 = 5’-CGCCACAAGGGCCATGAACAG-3’; TS3 = 5’-TAACATCATCATGAGACAGAGC-3’ and TS4 = 5’-CTCTGTTGTCTTAAACAAGAGA-3’), using the same cycling parameters as the first reaction.

DENV samples isolated by cell culture *in vitro* were further employed for differential DENV serotype diagnosis by indirect immunofluorescence using polyclonal antibodies to DENV serotypes (Biomanguinhos, FIOCRUZ, RJ, Brazil) as previously described (25).

### Quantification of Serum Soluble Mediators

Serum chemokines (CXCL8, CCL11, CCL3, CCL4, CCL2, CCL5, and CXCL10), pro-inflammatory cytokines (IL-1β, IL-6, TNF-α, IL-12, IFN-γ, IL-15, and IL-17), regulatory cytokines (IL-1Ra, IL-4, IL-5, IL-9, IL-10, and IL-13) and growth factors (FGF-basic, PDGF, VEGF, G-CSF, GM-CSF, IL-7, and IL-2) were quantified by a high-throughput microbeads array (Bio-Plex Pro™ Human Cytokine 27-plex Assay, Bio-Rad Laboratories, Hercules, CA, USA) according to the manufacturer’s instructions. Data acquisition was carried out on a Luminex 200 System using the Bioplex Manager Software. Final concentrations of serum soluble mediators were estimated by 5-parameter logistic regression according to the standard curve inserted on each experimental batch and the results expressed in pg/mL.

### Statistical Analysis

Data analysis was performed using GraphPad Prism v.9.1.1 software (San Diego, CA, USA). The Shapiro-Wilk normality test was conducted to assess data normality distribution. Considering the non-parametric distribution of the results, comparative analysis between DENV *vs* NI was performed by Mann-Whitney test. Multiple comparative analysis among subgroups according to DENV serotypes and days after symptom onset was carried out by Kruskal-Wallis test, followed by Dunn’s post-test. In all cases, significance was considered at p < 0.05.

Heatmap constructs were assembled using the heatmap.2 function in the R and gplots package (Project for Statistical Computing Version 3.0.1). The analysis was performed using customized functions available at Bioconductor packages, considering a Row Z-score, scaled from −2 to +2. Signatures of serum soluble mediators were constructed for NI, DENV, and DENV subgroups by first converting the serum levels of soluble mediators, originally expressed as continuous variables (pg/mL) into categorical data (proportion, %) using the global 3^rd^ tercile values of each soluble mediator as the cut-off to calculate the proportion of subjects above the cut-off edges. The following cut-offs were employed: CXCL8 = 5.68; CCL11 = 34.67; CCL3 = 3.64; CCL4 = 18.86; CCL2 = 51.61; CCL5 = 953.95; CXCL10 = 537.07; IL-1β = 0.45; IL-6 = 2.43; TNF-α = 16.26; IL-12 = 1.13; IFN-γ = 25.50; IL-15 = 102.75; IL-17 = 3.80; IL-1Ra = 303.94; IL-4 = 1.33; IL-5 = 5.90; IL-9 = 10.00; IL-10 = 11.12; IL-13 = 2.29; FGF-basic = 3.69; PDGF = 357.22; VEGF = 137.43; G-CSF = 34.62; GM-CSF = 1.61; IL-7 = 15.36; and IL-2 = 1.75. Thereafter, the proportions of subjects with increased levels of serum soluble mediators (above the 3^rd^ tercile) were calculated and the ascendant signatures built to underscore those molecules with proportion higher than 50%.

Correlation analysis was employed to construct integrative correlation matrices and assemble integrative serum soluble mediator networks. Pearson and Spearman correlation tests were used to obtain the significant “r” scores at p<0.05. The Cytoscape software platform (available at https://cytoscape.org) was employed to construct the complex networks with eccentric layout, with nodes representing the serum soluble mediators. Connecting edges illustrate weak/moderate (“r” scores between |0.1 to 0.66|) and strong correlations (“r” scores ≥ |0.67|) between pairs of attributes. Attributes without strong correlations were distributed in the network periphery. Serum soluble mediators presenting at least 5 strong correlations were assembled in the center with the number of serum soluble mediators and connections between them used to define the central connectivity.

The magnitude of change in the serum levels of soluble mediators was calculated as the proportion ratio according to the median values observed in non-infected healthy controls. Venn diagram analysis (http://bioinformatics.psb.ugent.be/webtools/Venn/) was carried out online to identify common and exclusive soluble mediators with altered levels observed in DENV serotypes.

## Results

### Overall Profile of Serum Soluble Mediators During Acute DENV Infection

**Figure 1:**
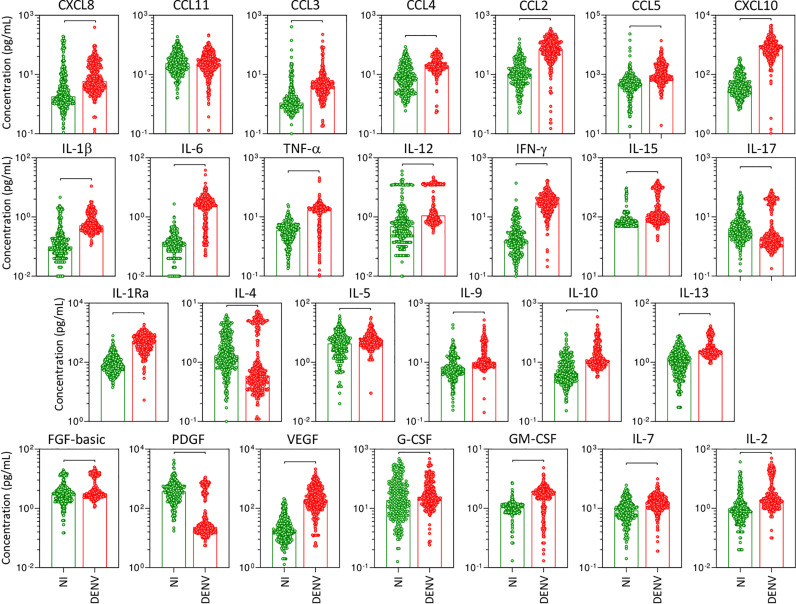
Overall profile of serum soluble mediators during acute DENV infection. The levels of chemokines (CXCL8, CCL11, CCL3, CCL4, CCL2, CCL5, CXCL10), pro-inflammatory cytokines (IL-1β, IL-6, TNF-α, IL-12, IFN-γ, IL-15, IL-17), regulatory cytokines (IL-1Ra, IL-4, IL-5, IL-9, IL-10, IL-13), and growth factors (FGF-basic, PDGF, VEGF, G-CSF, GM-CSF, IL-7, and IL-2) were measured in serum samples from patients with acute DENV infection – “DENV” (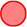 n=317), represented by red dots/bars on the right side of each panel, as compared to the reference non-infected healthy controls – “NI” (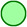 n=319), represented by green dots/bars on the left side of each panel. Quantification of serum soluble mediators was carried out by a high-throughput microbeads array as described in the Material and Methods section. The results are presented as the scattering of individual values expressed in pg/mL over bar plots highlighting the median on each bar. Significant differences at p <0.05 are indicated by connecting lines.

The panoramic analysis of chemokines (CXCL8, CCL11, CCL3, CCL4, CCL2, CCL5, CXCL10), pro-inflammatory cytokines (IL-1β, IL-6, TNF-α, IL-12, IFN-γ, IL-15, IL-17), regulatory cytokines (IL-1Ra, IL-4, IL-5, IL-9, IL-10, IL-13), and growth factors (FGF-basic, PDGF, VEGF, G-CSF, GM-CSF, IL-7, and IL-2) was performed in serum samples from patients with acute DENV infection as compared to the non-infected healthy controls (NI) and the results are shown in **Figure 1**. Overall, data analysis demonstrated that most chemokines, cytokines, and growth factors were increased in DENV patients as compared to NI. Conversely, lower levels of IL-17, IL-4, and PDGF were observed in DENV patients as compared to NI. No significant differences were observed in the serum levels of CCL11 (**Figure 1**).

### Panoramic Overview of Serum Soluble Mediators During Acute Infection With Distinct DENV Serotypes

**Figure 2:**
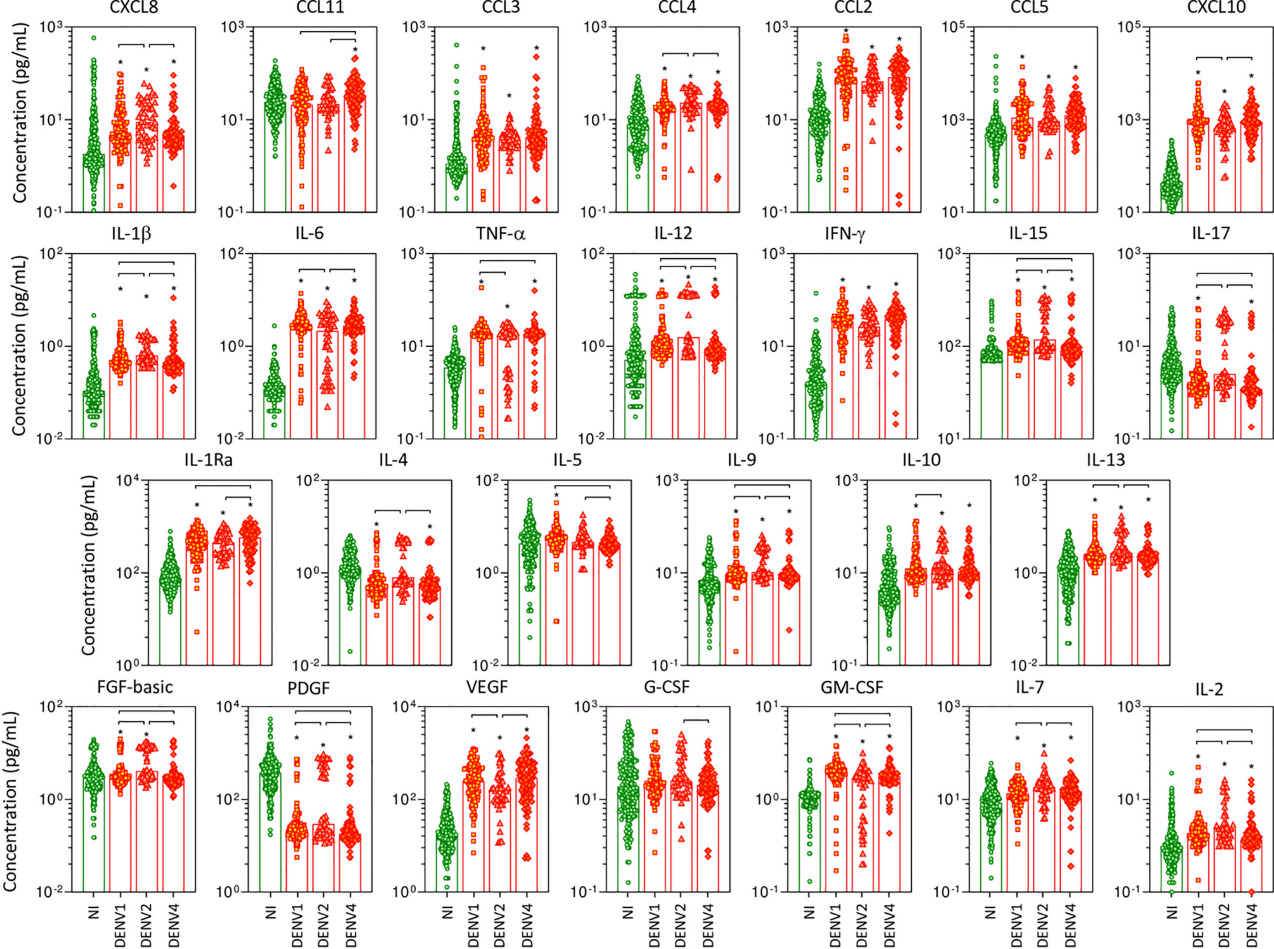
Panoramic overview of serum soluble mediators during acute infection with distinct DENV serotypes. The levels of chemokines (CXCL8, CCL11, CCL3, CCL4, CCL2, CCL5, CXCL10), pro-inflammatory cytokines (IL-1β, IL-6, TNF-α, IL-12, IFN-γ, IL-15, IL-17), regulatory cytokines (IL-1Ra, IL-4, IL-5, IL-9, IL-10, IL-13), and growth factors (FGF-basic, PDGF, VEGF, G-CSF, GM-CSF, IL-7, and IL-2) were measured in serum samples from patients with acute infection with distinct DENV serotypes (n=269), referred as: “DENV1” (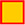 n=116), “DENV2” (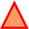 n=52), and “DENV4” (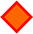 n=101), represented by symbols/bars on the right side of each panel, as compared to the reference non-infected healthy controls – “NI” (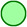 n=319), represented by green dots/bars on the left side of each panel. Quantification of serum soluble mediators was carried out by high-throughput microbeads array as described in the Material and Methods section. The results are presented as the scattering of individual values expressed in pg/mL over bar plots highlighting the median on each bar. Significant differences at p <0.05 are indicated by an asterisk (*), and connecting lines for comparisons with non-infected healthy controls and among DENV serotypes, respectively.

The overall snapshot of chemokines, pro-inflammatory cytokines, regulatory cytokines, and growth factors was performed in serum samples from patients with acute DENV infection, further classified according to the DENV serotypes as DENV1, DENV2, and DENV4, as compared to NI (**Figure 2**). Data analysis demonstrated that in general, regardless of the DENV serotype, the levels of the soluble factors were increased as compared to NI. Of note was the superior increase of several soluble mediators observed in DENV2 as compared to DENV1 and DENV4, including CXCL8, CCL4, IL-1β, IL-12, IL-15, IL-17, IL-4, IL-9, IL-13, FGF-basic, PDGF, IL-7, and IL-2. Conversely, lower levels of CXCL10, IL-6, VEGF, and GM-CSF were observed in DENV2 patients as compared to DENV1 and DENV4. Several changes were observed between DENV1 and DENV4, including increased levels of IL-1β, TNF-α, IL-12, IL-15, IL-5, IL-9, FGF-basic, GM-CSF, and IL-2 observed in DENV1, while increased levels of CCL11 and IL-1Ra along lower levels of IL-17 and PDGF were observed in DENV4 (**Figure 2**).

To further assess the magnitude of changes in serum levels of soluble mediators in patients with acute infection with distinct DENV serotypes, the fold change ratio was calculated for each sample according to the median values observed in NI (**Supplementary Figure 1**). Data analysis demonstrated that PDGF is universally identified with decreased levels (fold change <0.4) in all DENV serotypes as compared to NI. On the other hand, the levels of CXCL10, IL-6, and IFN-γ are substantially increased (fold change ≥10), regardless of the DENV serotypes. The analysis of soluble mediators with noteworthy increased levels (fold change ≥3-9) revealed that while CCL3, CCL2, IL-1β, TNF-α, and IL1-Ra were commonly observed in all DENV serotypes, CXCL8, CCL4, and IL-12 were elevated only in patients with DENV2 infections (**Supplementary Figure 1**).

### Heatmap Analysis and Signatures of Serum Soluble Mediators During Acute DENV Infection

**Figure 3:**
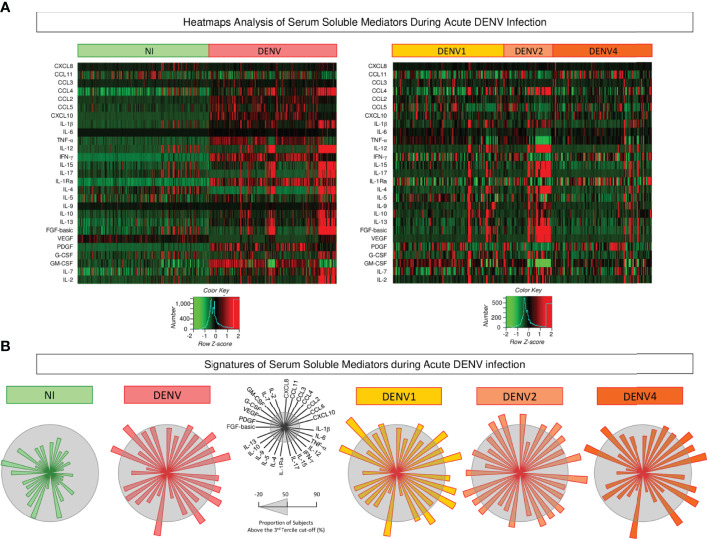
Heatmap analysis and signatures of serum soluble mediators during acute DENV infection. **(A)** Heatmap constructs of soluble mediators defined to cluster profiles of chemokines (CXCL8, CCL11, CCL3, CCL4, CCL2, CCL5, CXCL10), pro-inflammatory cytokines (IL-1β, IL-6, TNF-α, IL-12, IFN-γ, IL-15, IL-17), regulatory cytokines (IL-1Ra, IL-4, IL-5, IL-9, IL-10, IL-13), and growth factors (FGF-basic, PDGF, VEGF, G-CSF, GM-CSF, IL-7, and IL-2) observed in serum samples from patients with acute DENV infection – “DENV” (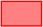 n=319), further classified according to the DENV serotypes (n=269) into: DENV1 (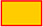 n=116), DENV2 (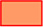 n=52), DENV4 (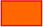 n=101) as compared to the reference non-infected healthy controls – “NI” (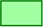 n=319). The soluble mediators were measured by a high-throughput microbeads array as described in the Material and Methods section. The individual levels of serum soluble mediators (attributes), expressed in pg/mL, were used to calculate the Z-score for each attribute and further generation of heatmap constructs. The Row Z-score scaled from −2 to 2 is illustrated as defined in the color key gradients provided in the Figure. **(B)** Overall signature of chemokines, pro-inflammatory cytokines, regulatory cytokines, and growth factors assembled for patients with acute DENV infection – “DENV”, further classified according to the DENV serotypes as: DENV1, DENV2, and DENV4 as compared to the reference non-infected healthy controls – “NI”. Signatures of serum soluble mediators were built as described in Material and Methods, by first converting the serum levels of soluble mediators, originally expressed as continuous variables (pg/mL) into categorical data (percentual, %) using the upper 3^rd^ tertile values of each serum mediator as the cut-off to identify the proportion of subjects above the cut-off edges. The final data are shown in a radar chart, with each axis representing one serum mediator. The 50^th^ percentile (gray zone) was used to underscore the serum soluble mediators with increased proportion (≥ 50%) in each study group.

Aiming at characterizing the global outlook of serum soluble mediators during acute DENV infection, heatmap constructs and soluble mediator signatures were assembled, and the data are presented in **Figure 3**. Overall, the heatmaps illustrate the massive release of most serum soluble mediators in DENV patients as compared to NI. Moreover, the heatmap constructs further confirmed the marked increase of several soluble mediators in DENV2 patients as compared to DENV1 and DENV4 (**Figure 3A**). The analysis of serum soluble mediator signatures assembled with categorical data further corroborates these findings. Using a general cut-off at the 50^th^ percentile, higher release of serum soluble mediators was observed in DENV (CCL3, CCL2, CCL5, CXCL10, IL-1β, IL-6, TNF-α, IFN-γ, IL-1Ra, VEGF, and GM-CSF), and lower PDGF as compared to NI. Moreover, the results also showed that DENV2 (CXCL8, CCL3, CCL4, CCL2, CXCL10, IL-1β, TNF-α, IL-12, IFN-γ, IL-15, IL-1Ra, IL-9, IL-10, IL-13, FGF-basic, VEGF, IL-7, and IL-2) displayed higher upregulation of serum soluble mediators as compared to DENV1 (CCL3, CCL2, CCL5, CXCL10, IL-1β, IL-6, TNF-α, IFN-γ, IL-15, IL-1Ra, VEGF, GM-CSF and IL-2), and DENV4 (CCL3, CCL4, CCL2, CCL5, CXCL10, IL-6, TNF-α, IFN-γ, IL-1Ra, VEGF, GM-CSF) (**Figure 3B**).

### Integrative Correlation Matrices and Networks of Serum Soluble Mediators During Acute DENV Infection

**Figure 4:**
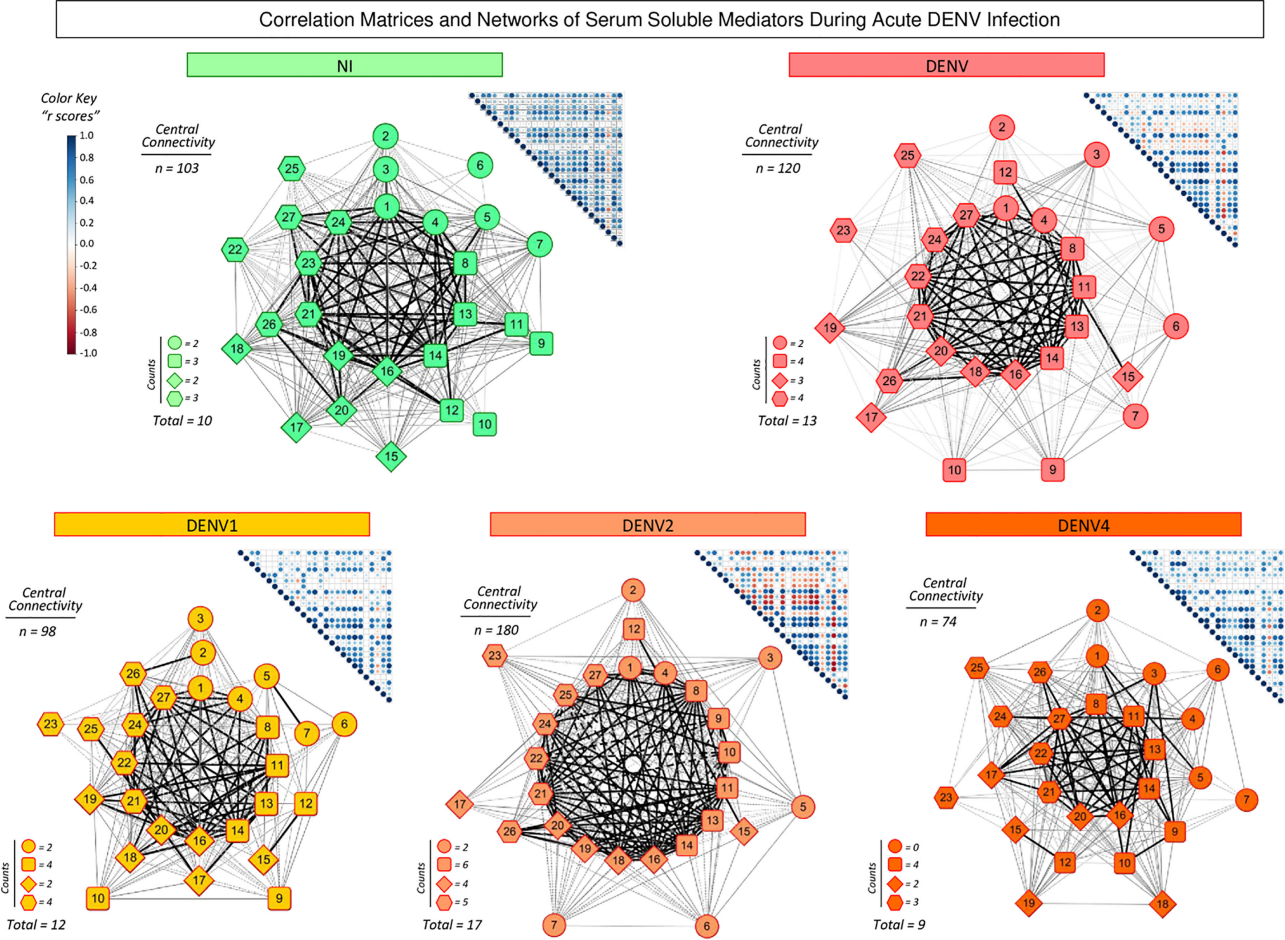
Integrative correlation matrices and networks of serum soluble mediators during acute DENV infection. Comprehensive correlation matrices were assembled based on the Pearson and Spearman “r” scores between chemokines, pro-inflammatory cytokines, regulatory cytokines and growth factors measured in serum samples from patients with acute DENV infection – “DENV” (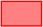 n=319), further classified according to the DENV serotypes (n=269) into: DENV1 (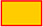 n=116), DENV2 (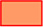 n=52), DENV4 (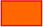
n=101) as compared to the reference non-infected healthy controls – “NI” (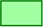 n=319). The soluble mediators were measured by a high-throughput microbeads array as described in the Material and Methods section. Panoramic correlation overviews are shown as triangle template matrices with each square intersection representing the correlation “r” score between pairs of attributes. The “r” scores of significant correlations (p< 0.05) are represented by circles of proportional sizes, scaled from −1 to +1 with a gradient for negative (red dots) and positive (blue dots) color keys. The white squares represent non-significant correlations. Networks were built using an eccentric layout based on significant correlations, with nodes representing the chemokines ( 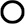 1=CXCL8; 2=CCL11; 3=CCL3; 4=CCL4; 5=CCL2; 6=CCL5 and 7=CXCL10), pro-inflammatory cytokines (8=IL-1β; 9=IL-6; 10=TNF-α; 11=IL-12; 12=IFN-γ; 13=IL-15 and 14=IL-17), regulatory cytokines (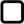 15=IL-1Ra; 16=IL-4; 17=IL-5; 18=IL-9; 19=IL-10 and 20=IL-13), and growth factors (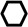 21=FGF-basic; 22=PDGF; 23=VEGF; 24=G-CSF; 25=GM-CSF; 26=IL-7; and 27=IL-2). Connecting edges illustrate weak/moderate (|0.1 to 0.66|, gradient gray thin lines) and strong “r” scores (≥ |0.67|, thick black lines) between pairs of attributes. Negative correlations are underscored by dashed lines. Attributes without strong correlations are distributed in the network periphery. Serum soluble mediators presenting at least 5 strong correlations are assembled in the center with the number of each category (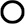, 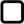, 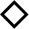 and 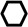), and connections between them (central connectivity) provided in the Figure.

To better understand the interrelationship between distinct serum soluble mediators during acute DENV infection, integrative correlation analysis was carried out and comprehensive matrices were created to assemble networks and calculate the neighborhood connectivity (**Figure 4**). Connectivity power networks were assembled using an eccentric layout based on the profile of significant correlation indices. The analysis of central connectivity scores, defined for serum soluble mediators with at least 5 strong correlations, revealed that acute DENV infection exhibited higher connectivity (n=120) involving more serum soluble mediators (n=13) as compared to NI (n= 103 and 10, respectively). In addition, the analysis of networks triggered by distinct DENV serotypes demonstrated that DENV2 displayed a more complex network, with higher central connectivity (n=180) enrolling more serum soluble mediators (n=17) as compared to DENV1 (n=98 and 12) and DENV4 (n=74 and 9) (**Figure 4**).

### Timeline Kinetics of Serum Soluble Mediators During Acute DENV Infection

**Figure 5:**
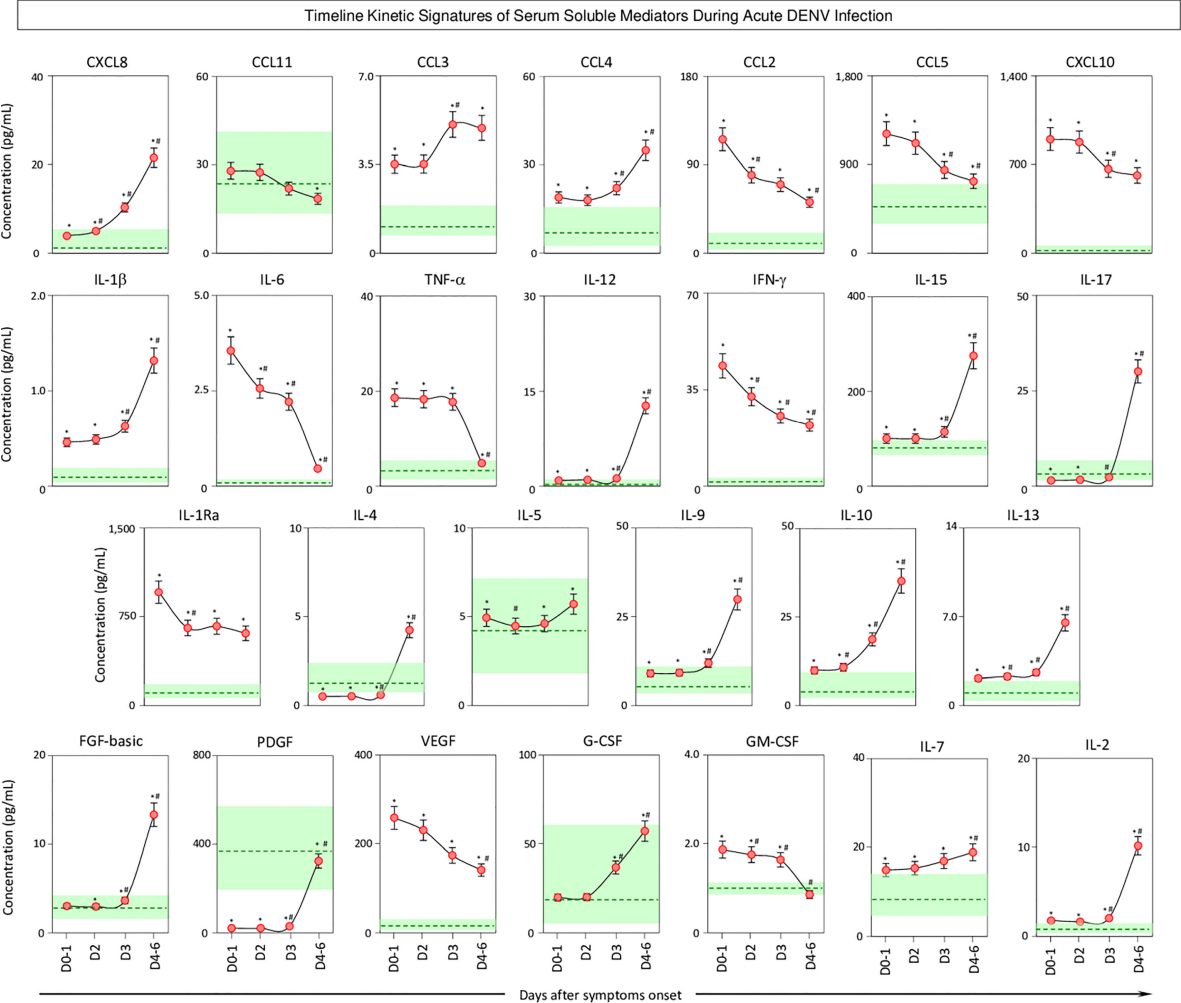
Timeline kinetics of serum soluble mediators during acute DENV infection. The dynamic kinetics of chemokines (CXCL8, CCL11, CCL3, CCL4, CCL2, CCL5, CXCL10), pro-inflammatory cytokines (IL-1β, IL-6, TNF-α, IL-12, IFN-γ, IL-15, IL-17), regulatory cytokines (IL-1Ra, IL-4, IL-5, IL-9, IL-10, IL-13), and growth factors (FGF-basic, PDGF, VEGF, G-CSF, GM-CSF, IL-2, and IL-7) was characterized in serum samples from patients with acute DENV infection (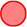 n=313) by cross-sectional analysis at four consecutive time points (Days = D), including: D0-1 (n=89), D2 (n=100), D3 (n=71), and D4-6 (n=53) after symptom onset. Measurements were carried out by a high-throughput microbeads array as described in the Material and Methods section. The results are presented as a line chart of median values (± 10% of median) at each time point along with the timeline kinetics. The green zone represents the reference interquartile range (25^th^-75^th^) observed for non-infected healthy controls “NI” (n=319). Significant differences at p<0.05 are identified by asterisks (*) for comparisons with “NI” and (#) for comparisons with the immediately preceding time-point.

The kinetics of serum soluble mediators in patients with acute DENV infection was carried out by cross-sectional analysis at four consecutive time points (Days = D), including D0-1, D2, D3, and D4-6 after symptom onset. The data are shown in **Figure 5**. Data analysis revealed several serum soluble mediators with a progressive increase along the timeline kinetics towards D4-6, including CXCL8, CCL3, CCL4, IL-1β, IL-12, IL-15, IL-17, IL-4, IL-5, IL-9, IL-10, IL-13, FGF-basic, PDGF, G-CSF, IL-7, and IL-2. On the other hand, a range of serum soluble mediators displayed a progressive decline towards D4-6, as observed for CCL11, CCL2, CCL5, CXCL10, IL-6, TNF-α, IFN-γ, IL-1Ra, VEGF, and GM-CSF (**Figure 5**).

### Timeline Kinetics Signatures of Serum Soluble Mediators During Acute Infection With Distinct DENV Serotypes

**Figure 6:**
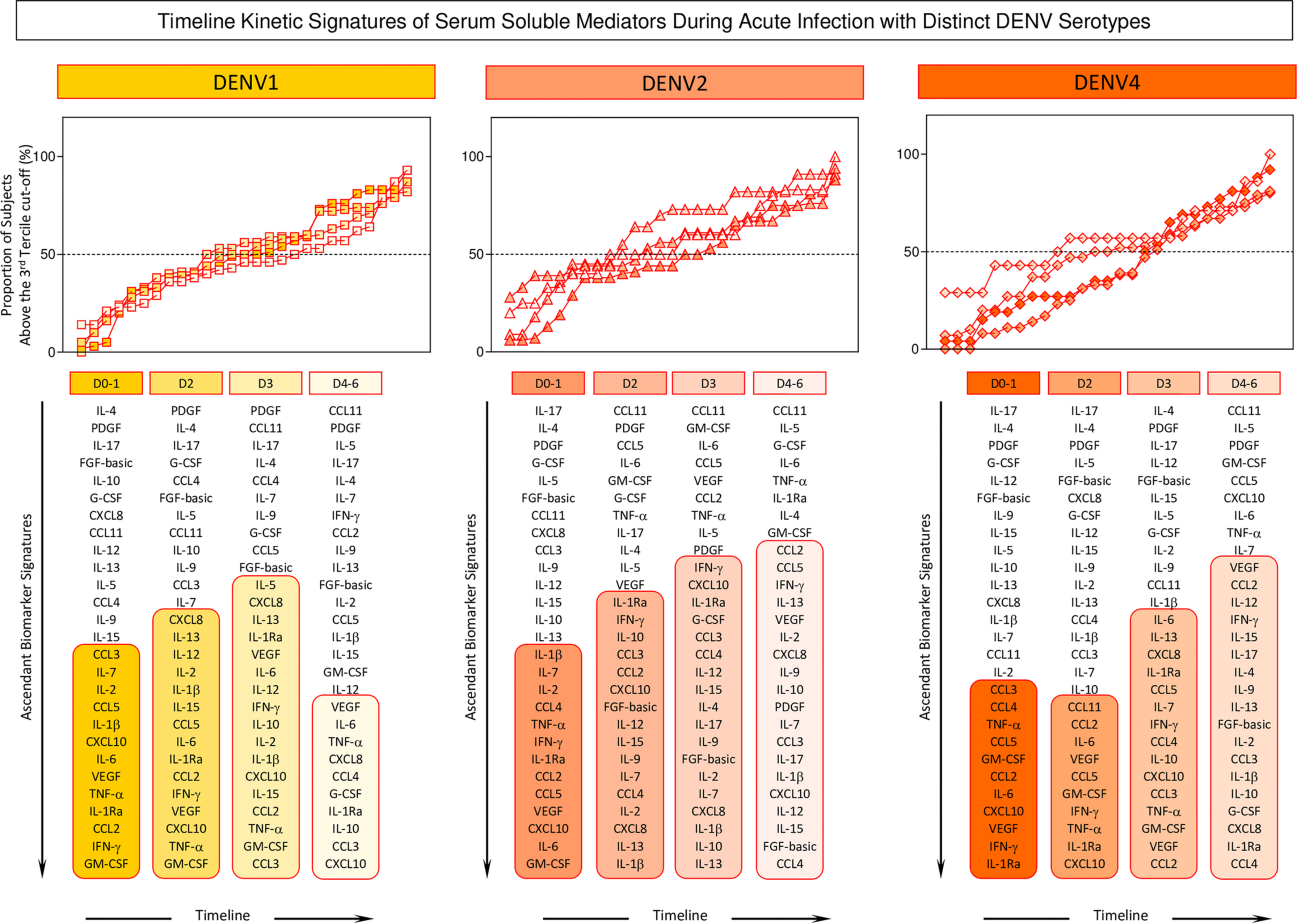
Timeline kinetics signatures of serum soluble mediators during acute infection with distinct DENV serotypes. Overall signature of chemokines (CXCL8, CCL11, CCL3, CCL4, CCL2, CCL5, CXCL10), pro-inflammatory cytokines (IL-1β, IL-6, TNF-α, IL-12, IFN-γ, IL-15, IL-17), regulatory cytokines (IL-1Ra, IL-4, IL-5, IL-9, IL-10, IL-13), and growth factors (FGF-basic, PDGF, VEGF, G-CSF, GM-CSF, IL-7 and IL-2) were assembled for patients with acute infection with distinct DENV serotypes (n=265), referred to as: DENV1 (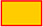 n=114), DENV2 (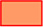 n=52), and DENV4 (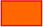 n=99) by cross-sectional analysis at four consecutive time points (Days = D), including: D0-1 (n=42; 17; 26), D2 (n=40; 18; 36), D3 (n=17; 11; 30) and D4-6 (n=15; 6; 7) after symptom onset. The serum soluble mediators were measured by a high-throughput microbeads array as described in Material and Methods. Signatures of serum soluble mediators were built by first converting the serum levels of soluble mediators, originally expressed as continuous variables (pg/mL) into categorical data (percentual, %) using the upper 3^rd^ tertile values of each serum mediator as the cut-off to identify the proportion of subjects above the cut-off edges. The final data are shown as line charts displaying the serum soluble mediator signature at each time point. Column diagrams are provided below to underscore the upregulated soluble serum mediators along the timeline kinetics. The 50^th^ percentile was used to underscore the serum soluble mediators with an increased proportion (≥ 50%) in each study group.

To further characterize the dynamic changes of soluble mediators in acute DENV1, 2, and 4 infection, chemokines, growth factors, and pro-inflammatory and regulatory cytokines were quantified at four consecutive time points (D0-1, D2, D3, and D4-6) after symptom onset (**Figure 6**). DENV1 infection showed a progressive increase of soluble mediators, with a peak at D3 and a decline towards D4-6. DENV2 infection showed a progressive increase of soluble mediators towards D4-6. Interestingly, DENV4 infection showed an early plateau followed by a late upregulation at D3 towards D4-6.

## Discussion

The pathogenesis of DENV infection involves a complex interaction between viral and host features. Risk factors for severe disease may include viral serotype, host age, genetic background, and immunological profiles (4–6). In this sense, distinct DENV serotypes leading to diverse immune responses (9) may impact the disease outcome towards severe clinical forms (10). Despite the relevance of the DENV serotype association with higher rates of severe clinical forms of DENV infections (3, 8), the precise mechanism and the specific elements with which the immune response participates in this phenomenon are not completely elucidated.

From an immunological point of view, previous studies have already demonstrated that acute DENV infection elicits a massive release of several immunological mediators, comprising a high level of chemokines and pro-inflammatory cytokines (18, 24). It is strongly believed that soluble mediators play a key role in orchestrating the immune response against viral patterns. Therefore, there is a consensus that the storm of chemokines, cytokines, and growth factors may directly shape the immunopathogenesis of acute DENV infection (26–28). However, distinct studies report conflicting results, about the increased levels of soluble mediators (11–16). These discrepancies among different studies are probably associated with the study design including the time of sampling, the laboratory methods, and instruments employed to measure soluble mediators, and the DENV serotype causing infection.

Aiming at overcoming these drawbacks, the present investigation encompassed a detailed analysis of serum soluble mediators using a sensitive high-throughput microbeads array and took into consideration the DENV serotype causing infection as well as the time for symptom onset.

Our results demonstrate that distinct patterns and characteristic behavior of serum soluble mediators occurred during acute DENV infection, with DENV serotype-associated particularities as characterized by distinct profiles, rhythms, and dynamic network connectivity. The three serum soluble mediators CXCL10, IL-6, and IFN-γ were underscored as universal, regardless of the DENV serotype, with a substantial increase of more than 10 times the control samples. At the opposite extreme, PDGF appeared as the least expressed soluble mediator, with less than 0.4 times the control, regardless of the serotype. PDGF was originally found as a constituent of platelets and the overall universal decrease in this biomarker may be associated with the canonical thrombocytopenia observed in Dengue patients.

The chemokine system appears to play a role during DENV infection. It has been shown that CXCL10 production improves host resistance, controlling the viral replication during DENV infection (29). It has also been shown that IL-6, produced by macrophages and activated endothelial cells, is a major mediator of fever and acute-phase reactions, and is produced by macrophages and activated endothelial cells. Furthermore, IL-6 mediates changes in coagulation and fibrinolysis by inducing the expression of tissue factors by endothelial cells (30). IL-6 production has been linked to several systemic changes in an acute inflammatory response (31), and the overproduction of IL-6 mediated by different DENV serotypes plays an important role in the pathogenesis triggered by the dengue virus (32). Moreover, increased serum levels of IL-6 have been reported in patients with severe DENV infections (33). In agreement with our findings, previous studies have already shown that IFN-γ is highly produced during DENV infection. IFN-γ is a pro-inflammatory cytokine that controls the production of nitric oxide and has an important antiviral role (34). Increased levels of IFN-γ are directly associated with protection against fever and high viremia, and with higher survival rates in patients with hemorrhagic signs (29, 34, 35). In agreement with that, IFN-γ-deficient mice are more susceptible to DENV2 and DENV3 infection (36, 37). It has been previously reported that lower levels of PDGF are observed in thrombocytopenic DENV patients, suggesting the relevant role of this growth factor in the pathogenesis of DENV (38). Overall, together with previous reports, our data suggest that the profile of CXCL10, IL-6, IFN-γ and PDGF may represent a useful set of biomarkers to monitor DENV outcome caused by distinct serotypes with a putative prognostic purpose. It is important to mention that other febrile illness may also lead to similar profile of serum soluble mediators as observed during the acute DENV infection (39, 40). In this sense, we have already reported that patients with acute ZIKV and CHIKV infection presented enhanced levels of serum soluble mediators including CXCL10 and IL-6. Future studies are still required to further address this issue, comparing a wide range of soluble mediators in distinct acute febrile illnesses in addition to the comparison with healthy controls.

Of note, our data have demonstrated that the profile of circulating soluble mediators differs substantially during acute DENV infection with distinct serotypes. Specifically, CXCL8, CCL4, and IL-12 were selectively increased in DENV2 as compared to DENV1 and DENV4. It has been shown that together with IL-6, CXCL8 mediates the derangement of coagulation and fibrinolysis acts and synergistically in the upregulation of tissue factors by endothelial cells (30). Clinical studies in endemic areas have described a correlation between DENV disease outcome and the levels of CCL4 which are intimately related to hypotension, thrombocytopenia, and hemorrhagic shock (14, 41). In general, the studies demonstrated that IL-12 seems to play a protective role during DENV-2 infection, preceding IFN-γ production (36). It has been previously suggested that lower levels of IL-12 are observed in patients with severe hemorrhagic DENV infection (42).

Clearly, there is a substantial redundancy between soluble mediator functions (i.e., the lack of one may be compensated by another with overlapping activities), and it has been previously demonstrated that the same soluble mediator may play distinct roles depending on the immunological microenvironment. In this sense, intending to perform an integrative analysis of chemokines, cytokines, and growth factors, we have constructed a comprehensive landscape of networks to illustrate the panoramic interplay between them. Our findings showed that DENV infection exhibited higher connectivity between more soluble mediators with DENV2 displaying a more complex network.

Regardless of the substantial number of patients enrolled, the present study has some limitations, such as: lack of previous studies on soluble mediators of DENV infection with distinct serotypes for comparative analysis; observational design of multiple comparisons without corrections for co-morbidities or other confounding variables; and lack of information or limited access to clinical records to allow data analysis according to disease severity and outcome to recovery or death. It is well known that secondary infection, regardless of DENV serotype, is associated with more severe disease as compared to primary infection. Moreover, although we have applied distinct laboratory methods to diagnose acute DENV infection, ascertaining whether the cases represent the primary or secondary dengue episode was not possible. Furthermore, the qPCR data were available for a small number of patients which did not allow an accurate and representative measurement of viremia neither to carry out a comparative analysis between viremia and levels of serum soluble mediators. Additional studies are currently in progress to access the impact of aging on the immunological profile of patients infected with distinct DENV serotypes.

Dengue is a disease that has annual epidemic peaks at different intensities and is related to different DENV serotypes. The reintroduction of DENV-4 in 2010 in Brazil, and the establishment of multiple co-circulating serotypes in different regions of the country are factors that may justify the non-predominance of DENV-3, given that in previous years, this serotype was predominant (43).

Altogether, these findings may provide novel insights to subsidize the understanding of DENV pathogenesis caused by distinct serotypes, highlighting useful biomarkers for future applications to predict severe disease outcomes and establish therapeutic interventions.

## Data Availability Statement

The raw data supporting the conclusions of this article will be made available by the authors, without undue reservation.

## Ethics Statement

The studies involving human participants were reviewed and approved by Ethical Committee at Instituto René Rachou/Fundação Oswaldo Cruz (FIOCRUZ-Minas) – Belo Horizonte, Minas Gerais, Brazil (CAAE 41591015.2.0000.5091), and Fundação de Medicina Tropical Dr. Heitor Vieira Dourado, Manaus, Amazonas, Brazil (CAAE 80866017.8.0000.0005). Written informed consent to participate in this study was provided by the participants’ legal guardian/next of kin.

## Author Contributions

Designing research study: MB, LR, PV, OM-F, MA, MSF, and LM. Advisory Committee: KB, VP-M, AC-A, JC-d-R, CG, and AC. Funding Acquisition: AT-C, MB, LR, PV, and OM-F. Conducting experiments and acquiring data: MC-d-S, PS, AC-A, VR, FM, JS-A, IC-R, VS, ESF, EM, ECSF, EVPS, BR, ÉBS, and MNOF. Analyzing data: MC-d-S, PS, AC-A, JB-d-S, MG, LRA, OM-F, MA, and MSF. Writing and reviewing the manuscript: MC-d-S, PS, VP-M, MR, JC-d-R, LRVA, CG, MB, AC, PV, OM-F, MA, MSF, and LM. All authors contributed to the article and approved the submitted version.

## Funding

The study was supported by the Conselho Nacional de Desenvolvimento Científico e Tecnológico - CNPq. Funding was also obtained from Fundação de Amparo à Pesquisa do Estado do Amazonas (FAPEAM/PPP-CNPq, EDITAL N. 016/2014), Ministério da Saúde do Brasil (Chamada Pública n° 01/2012, Convênio # 776823/2012) and INCT para Febres Hemorrágicas Virais (INCT-FHV - 573739/2008-0).

## Conflict of Interest

The authors declare that the research was conducted in the absence of any commercial or financial relationships that could be construed as a potential conflict of interest.

## Publisher’s Note

All claims expressed in this article are solely those of the authors and do not necessarily represent those of their affiliated organizations, or those of the publisher, the editors and the reviewers. Any product that may be evaluated in this article, or claim that may be made by its manufacturer, is not guaranteed or endorsed by the publisher.
