# Supplementary material for: Serum Soluble Mediator Profiles and Networks During Acute Infection With Distinct DENV Serotypes
Source: Front Immunol. 2022 May 31;13:892990. doi: 10.3389/fimmu.2022.892990 (PMC9193801; doi:10.3389/fimmu.2022.892990)
Supplement: Supplementary file 1 [file Table_1.docx]

Supplementary Material

**Supplementary Figure 1.** *Magnitude of changes in serum soluble mediators during acute infection with distinct DENV serotypes*. The magnitude of change in the levels of chemokines, pro-inflammatory cytokines, regulatory cytokines, and growth factors was calculated for individual samples from patients with acute infection with distinct DENV serotypes (n=269), referred to as DENV1 (, n=116), DENV2 (, n=52), DENV4 (, n=101). Measurements of soluble mediators were carried out by Luminex Bio-plex platform as described in the Material and Methods section. The fold change values were calculated as the proportion ratio according to the median values observed in non-infected healthy controls. The results are expressed as median fold change ratio and presented in bar chart format as ascendant fold change profiles. The soluble mediators with decreased levels (<0.4), increased levels (≥3-9), and increased levels (≥10) were underscored (bottom color rectangles). Venn diagram analysis was carried out to identify common and exclusive soluble mediators with altered levels observed in DENV1, DENV2, and DENV4.
